# Identification of Potential Biomarkers and Metabolic Pathways of Different Levels of Heat Stress in Beef Calves

**DOI:** 10.3390/ijms231710155

**Published:** 2022-09-05

**Authors:** Won-Seob Kim, Jongkyoo Kim, Hong-Gu Lee

**Affiliations:** 1 Department of Animal Science and Technology, Sanghuh College of Life Science, Konkuk University, Seoul 05029, Korea; 2 Department of Animal Science, Michigan State University, East Lansing, MI 48824, USA; 3 Animal Science and Food Science and Human Nutrition, Michigan State University, East Lansing, MI 48824, USA

**Keywords:** biomarker, energy metabolism, heat stress, metabolic regulatory pathways, molecular mechanisms

## Abstract

Heat stress (HS) damages the global beef industry by reducing growth performance causing high economic losses each year. However, understanding the physiological mechanisms of HS in Hanwoo calves remains elusive. The objective of this study was to identify the potential biomarkers and metabolic pathways involving different levels of heat stress in Hanwoo calves. Data were collected from sixteen Hanwoo bull calves (169.6 ± 4.6 days old, BW of 136.9 ± 6.2 kg), which were maintained at four designated ranges of HS according to the temperature–humidity index (THI) including: threshold (22 to 24 °C, 60%; THI = 70 to 73), mild (26 to 28 °C, 60%; THI = 74 to 76), moderate (29 to 31 °C, 80%; THI = 81 to 83), and severe (32 to 34 °C, 80%; THI = 89 to 91) using climate-controlled chambers. Blood was collected once every three days to analyze metabolomics. Metabolic changes in the serum of calves were measured using GC-TOF-MS, and the obtained data were calculated by multivariate statistical analysis. Five metabolic parameters were upregulated and seven metabolic parameters were downregulated in the high THI level compared with the threshold (*p* < 0.05). Among the parameters, carbohydrates (ribose, myo-inositol, galactose, and lactose), organic compounds (acetic acid, urea, and butenedioic acid), fatty acid (oleic acid), and amino acids (asparagine and lysine) were remarkably influenced by HS. These novel findings support further in-depth research to elucidate the blood-based changes in metabolic pathways in heat-stressed Hanwoo beef calves at different levels of THI. In conclusion, these results indicate that metabolic parameters may act as biomarkers to explain the HS effects in Hanwoo calves.

## 1. Introduction

Temperature and humidity above the thermal neutral zone may increase the likelihood of heat stress (HS), resulting in animals’ physiological changes [1,2]. HS can impact the growth, lactation, reproduction, and welfare of most livestock animals such as beef [2,3], dairy [1,4], and poultry [5]. Among them, beef cattle are especially vulnerable to HS because they lack the ability to dissipate heat sufficiently [6,7]. Behavior and metabolic changes have been observed to maintain homeothermy in beef cattle, often at the expense of productivity and profitability [7,8]. External indicators such as heart rate, respiration rate, core body temperature, and behavior are commonly used to determine the damage caused by thermal stress [2,3,9]. However, these traditional parameters are often indirect and variable depending on the animal’s physical status and environment. Understanding metabolic mechanisms are essential, as they regulate homeostasis through various metabolic pathways in the body to overcome HS prior to external reactions.

Metabolomics analysis aids in the understanding of an organism’s overall metabolic map and functional regulation through the qualitative and quantitative analysis of metabolites [10]. Metabolomics has widely been used to predict the risk of disease and biomarkers and pathways in some metabolic disorders of cattle [11,12]. Measuring the total metabolic profile can be a robust and direct method that reflects various factors affecting animals’ bodies, such as feed, genetics, physiological, and environmental changes [7,13]. Through mining metabolic pathways such as carbohydrate, amino acids (AAs), and lipid metabolism, it is possible to systematically evaluate the physiological status, stress, and nutrients of cattle according to their diseases through a metabolic approach [14]. Given this review, metabolomics analysis offers a strong platform for the identification in both animals and humans of the pathophysiological alterations resulting from exposures to specific environmental stimuli [11].

The correlation between HS and metabolomics in milk and urine was reported in earlier investigations of dairy cows [15,16]. Additionally, it has been observed that due to decreased feed intake and alterations in a number of physiological parameters, HS had an impact on the metabolism of carbohydrates, AAs, and lipid in beef cattle [12,17] and dairy cows [14,16,18]. According to earlier research with Hanwoo calves, HS can have serious impacts on physiological parameters and growth performance [2,3,19]. To the best of our knowledge, no study has been conducted to investigate the metabolic changes associated with HS during a growing stage (5 to 6 months old).

In addition, most previous studies have simply measured the effect of normal versus severe HS, while there is a lack of research on how various levels of HS affect blood metabolites and pathways. Hence, we hypothesized that different levels of HS conditions could have varying effects on blood metabolic parameters and pathways in beef calves. Therefore, the objectives of the current study were to evaluate the biomarkers and related metabolic pathways during different levels of HS in Hanwoo calves.

## 2. Results

### 2.1. Metabolic Profiles

**Figure 1:**
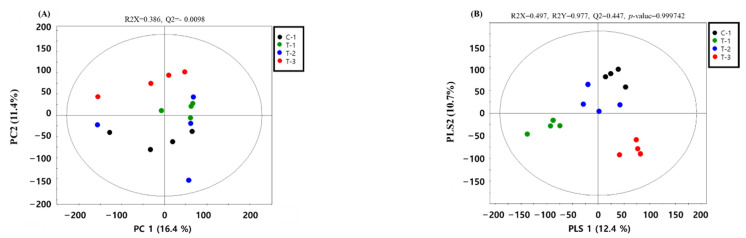
PCA (principal component analysis) score plot of each group in serum (**A**). PLS-DA (partial least squares discriminant analysis) score plot and isolate metabolic parameters (VIP > 0.7) of each group in serum (**B**).

Metabolic raw data from the serum were collected and analyzed through multivariate statistical analysis. The status of the metabolites in the group was determined in the score plot for the principal component analysis (PCA) obtained from serum (Figure 1). The distribution of the metabolite parameters in serum (R^2^X = 0.497, R^2^Y = 0.977, Q^2^ = 0.447, *p* = 0.9997) showed a clear separation in the partial least squares discriminant analysis (PLS-DA) model (Figure 1).

### 2.2. Detection and Identification of Metabolic Markers

**Figure 2:**
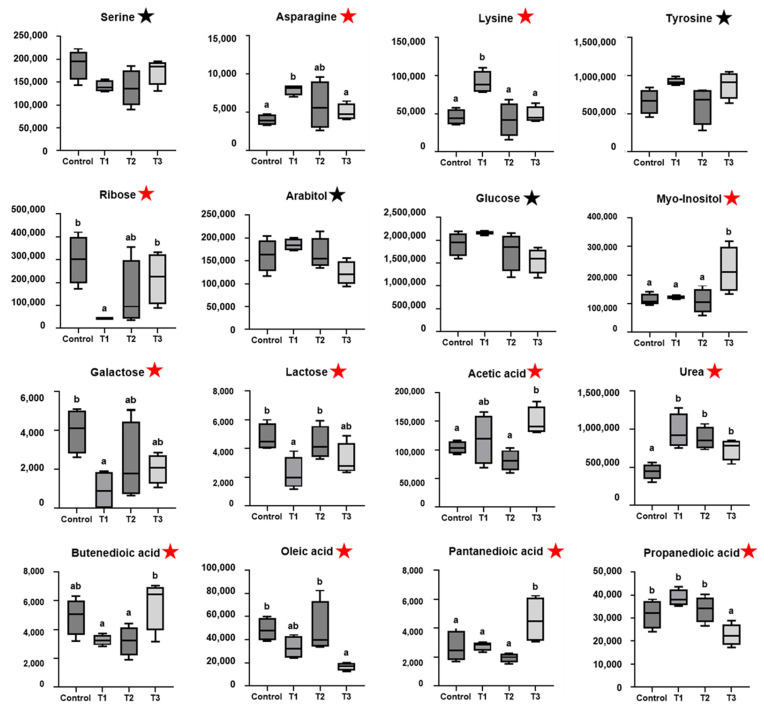
Metabolomics parameters (*n* = 4) in serum (Control, T1, T2, T3; VIP > 0.7, *p* < 0.05 (★), *p* < 0.1 (★). ^a,b^ Means with different superscripts differ significantly in each group (*p* < 0.05) based on Tukey’s test.

Detection and identification of metabolic biomarkers were based on the obtained results from the databases of the human metabolome database (HMDB) (http://www.hmdb.ca/), PUBCHEM (http://pubchem.ncbi.nlm.nih.gov/), and the Kyoto Encyclopedia of Genes and Genomes (KEGG) (http://www.genome.jp/gegg/, accessed on 11 August 2021). According to the PLS-DA analysis, 38 potential metabolic markers were separated from the serum based on a value of importance in the projection higher than 0.7 (variable important in projection; VIP > 0.7) (Table 1). In the results from the serum samples, asparagine (*p* = 0.040) and lysine (*p* = 0.002) in the mild (T1) group were significantly higher than those in the threshold (CON) group. Moreover, ribose (*p* = 0.030), galactose (*p* = 0.047), lactose (*p* = 0.028), urea (*p* = 0.003), butenedioic acid (*p* = 0.028), serine (*p* = 0.086; tendency) in the T1 group were significantly lower than those in the CON group (Figure 2). The myo-inositol (*p* = 0.018), acetic acid (*p* = 0.027), pentanedioic acid (*p* = 0.017), and tyrosine (*p* = 0.066; tendency) were found to be significantly increased in the severe (T3) group over those in the CON group. Oleic acid (*p* = 0.018), propanedioic acid (*p* = 0.006), glucose (*p* = 0.056; tendency), and arabitol (*p* = 0.059; tendency) in the T3 group were significantly lower than those in the CON group (Figure 2). Interestingly, we found that twelve metabolic parameters were related to HS.

### 2.3. Characterization and Functional Analysis of Metabolic Pathways in Serum

**Figure 3:**
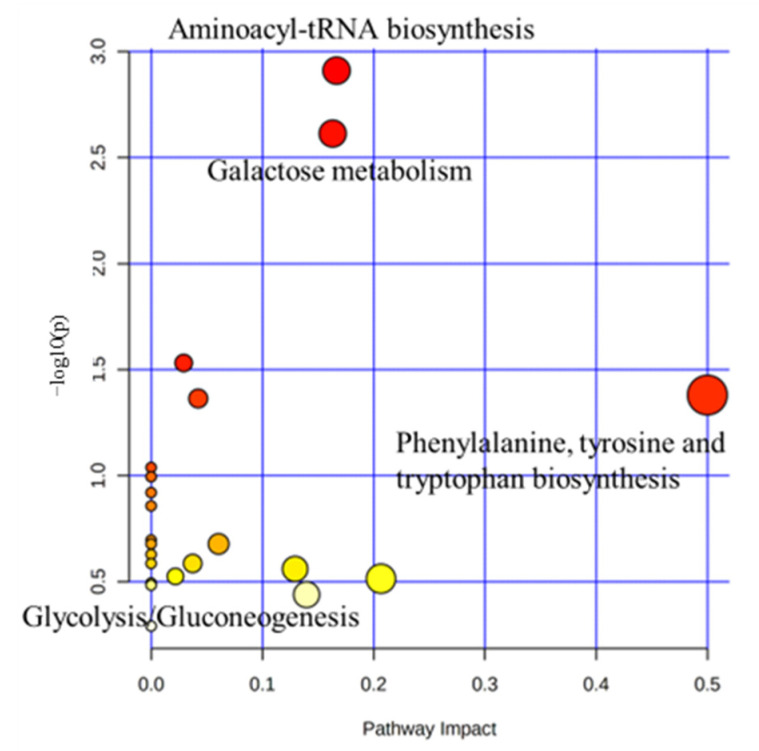
The metabolic pathway map related to metabolic profiling in serum, according to the effects of heat stress in each group. The *x*-axis represents the pathway impact values computed from pathway topological analysis, and the *y*-axis represents the −log of *p*-value obtained from pathway enrichment analysis. The pathways that were most significantly changed are characterized by both a high −log(*p*) value and high impact value (top right region). The color and size of each dot were associated with the −log(*p*) value and pathway impact value, respectively, where a small *p* value and high pathway impact value indicate the pathway is greatly influenced by heat stress.

Metabolic pathways were defined with the online MetPa system (METABOANALYST 4.0, http://www.metaboanalyst.ca/, accessed on 11 August 2021). Metabolites with significant changes in serum were imported into the metaboanalyst online system to generate the metabolome view list. The *Bos taurus* was selected for pathway analysis in the model organism interface. For the pathway enrichment analysis, an over-representation analysis was used. The pathway topology analysis was used on the relative betweenness centrality measure in the established metabolic network to predict the importance of metabolites. Potential targets were selected based on both impact value (not below 0.1) and *p*-value (no more than 0.05). Figure 3 shows the metabolome maps of the relevant metabolic pathways in the serum based on the KEGG database, respectively [20,21,22]. Potential function pathways are summarized in Table 2. The heat stressed group had significant changes of phenylalanine, tyrosine, and tryptophan biosynthesis (*p* = 0.042, impact = 0.50), aminoacyl-tRNA biosynthesis (*p* = 0.001, impact = 0.17), and galactose metabolism (*p* = 0.002, impact = 0.16), glyoxylate and dicarboxylate (*p* = 0.043, impact = 0.04), and glycolysis/gluconeogenesis (*p* = 0.029, impact = 0.03). However, no significant change was found in inositol phosphate metabolism (*p* = 0.317, impact = 0.13), pyruvate metabolism (*p* = 0.210, impact = 0.06), the phosphatidylinositol signaling system (*p* = 0.260, impact = 0.04), cysteine and methionine metabolism (*p* = 0.299, impact = 0.02), glycine, serine, threonine metabolism (*p* = 0.306, impact = 0.21), and tyrosine metabolism (*p* = 0.364, impact = 0.14).

## 3. Discussion

Reduced growth performance is one of the major responses noted in animals exposed to HS mainly due to the reduced feed intake. Our previous research showed that HS had adverse effects on a number of metrics including growth performance and physiological parameters in Hanwoo calves [3]. It has negative impacts on how nutrients including carbohydrates, proteins, and fats are metabolized. The metabolomic approach has not been used in Hanwoo cattle to see the observed nutritional metabolism pathways under HS conditions. The current study was conducted to interpret various phenomena in relation to metabolism under different levels of HS in Hanwoo calves. Twelve potential metabolite biomarkers, including pathways related to carbohydrate, AAs, and lipid metabolism, were identified.

HS raises the core body temperature of cattle; it causes an acclimation response to reduce the heat generated during digestion, which leads to a decrease in feed intake [13]. Previous studies reported that reduced feed intake due to HS conditions decreased cattle blood glucose and carbohydrate metabolites, affecting glycolysis and gluconeogenesis pathways [3,7,23]. In the present study, serum glucose, galactose, lactose, ribose, and arabitol were decreased in the mild and severe HS groups compared to the CON. Moreover, myo-inositol and acetic acid were significantly higher in the severe HS group than in the CON group. These changes in blood carbohydrate metabolites were seemingly associated with HS and its effects on glucose metabolism. A decreased serum glucose level was detected in the current study when calves were exposed to HS. This may be mainly due to the reduced feed intake under HS conditions. Since glucose is the primary form of energy transfer in the animal body, maintaining serum glucose is critical. A well-described mechanism of a glucose-sparing effect is that growing animals utilize fat or protein sources when on a lower nutritional plane or when in a negative energy balance [7].

Consequently, the lower glucose caused by HS can alter the glycolysis-related pathway and subsequent carbohydrate metabolism. Lactose consists of a galactose molecule and a glucose molecule [24]. Because fewer sources can synthesize lactose in HS circumstances, our finding showed that lactose showed a similar pattern of decreasing with galactose with a decline in blood glucose levels. Glucose is a form of sugar that is stored intracellularly as glycogen, whereas galactose is converted to glucose and then utilized, which can be oxidized in the process or stored as glycogen.

Inositol is one of the glycolytic polyalcohol that produces a stereoisomeric form capable of nine epimerization of hydroxyl groups [25]. It has been reported that myo-inositol is a modulator of insulin regulation and glucose homeostasis in animal models of insulin resistance [26]. When blood glucose and galactose levels decreased under the HS condition, myo-inositol synthesis increased in the body due to the regulation of homeostasis. In the present study, blood ribose was decreased when exposed to long-term HS conditions in Hanwoo calves. Ribose, a 5-phosphate ester, is usually produced from blood glucose by the pentose phosphate pathway. A previous study revealed that in cattle exposed to long-term HS, glucose utilization, rather than synthesizing as lactose, induced conversion to the pentose phosphate pathway and glycolysis, affecting energy production [27].

HS increases protein breakdown and AA mobilization, enabling further energy production for maintenance and growth [7]. Under stressful conditions, such as HS, circulating glucocorticoid levels are increased, which in turn reduces the rate of protein synthesis and improves proteolysis to generate AAs to serve as precursors for hepatic gluconeogenesis [28]. These results are consistent with the previous reports that blood cortisol levels were increased with exposure to HS in Hanwoo calves [2,3]. These phenomena produce higher glucogenic AAs to synthesize glucose according to protein breakdown, particularly under HS conditions while the glucose is limited. Glucogenic AAs support glucose and energy homeostasis in heat-stressed cattle [29]. Our data indicated that serum glucogenic AAs, including asparagine and tyrosine, were increased in the HS compared to the CON group. Glucose requirements during HS condition can be partially met by gluconeogenesis utilizing AA [29,30]. 

It is also noted that serum lysine and urea levels were greater in the HS group than that of the CON group. Lysine is one of the most limiting AAs for growing cattle and helps the protein synthesis required for skeletal muscle growth in calves [31,32]. Given that HS is likely to affect rumen fermentation processes, the HS-induced rise in blood urea nitrogen [7,33] may be the result of ineffective rumen microbial nitrogen uptake. In addition, a previous study [7] stated that increased blood urea nitrogen could be a downstream byproduct of skeletal muscle proteolysis in heat-stressed growing calves. A previous study reported that milk protein synthesis was more susceptible to HS than other components of milk, and HS caused nitrogen partitioning from milk protein to milk urea in dairy cows [34]. Consistently, Tian et al. [35] reported that HS elevated mobilization of AAs and increased the formation of urea in the HS group. These results demonstrated that HS induced a shift in the nitrogen metabolic pathway in dairy cows. Unknown factors such as skeletal-muscle protein breakdown or excessive rumen ammonia production may be the reasons behind the rise in blood urea nitrogen [36]. In agreement with the previous study, our results indicated that HS might induce migration of the nitrogen metabolic pathways in growing calves. 

HS is considered a catabolic signal and increases circulating cortisol which generally stimulates lipolysis and adipose triglyceride mobilization [3,4,37]. In addition, blood epinephrine has been used in many situations as an indicator to stimulate fat metabolism [38]. A previous study reported that under stressful conditions whole-body lipolysis increased but fatty acid oxidation decreased [39]. During HS, decreased *β*-oxidation largely utilized glycogen as an energy source rather than lipids to reduce heat generation [40]. In the current study, we found that serum levels of free fatty acids such as oleic acid were lower in the HS group compared to the CON. These findings indicate that HS induces overall changes in lipid metabolism via the reduction in *β*-oxidation in growing calves. 

HS affects the microbial environment of the rumen, causing an increase in pathogenic microorganisms, a decrease in adaptation pathways to the environment, and impaired immune responses and metabolic pathways [41]. Reduced feed intake and possible changes in rumen metabolism during HS led to a reduction in total and individual volatile fatty acid concentrations in rumen fluid [42]. Of these volatile fatty acids, acetic acid is an important substrate that has a strong impact on the expression of enzymes related to muscle growth [43]. The current study showed that the serum level of acetic acid was increased regardless of a decrease in feed intake under severe HS. In addition, short-chain fatty acids (fumaric acid and glutaric acid) showed a similar trend to acetic acid except for malonic acid. In general, acute feed restriction in stressful situations such as HS causes negative energy balance, alters rumen microbial composition, and reduces short-chain fatty acids [4]. However, it has been reported that short-chain fatty acids may increase due to adaptation to energy supply and homeostatic regulation during prolonged chronic HS [44]. These short-chain fatty acids can be utilized to produce energy via the TCA cycle. In addition, there was a previous study that showed a decrease in the dietary forage–concentrate ratio under feed restriction affected the population of rumen microorganisms and increased dry matter digestibility and short-chain fatty acids [45,46,47]. These findings are supported by the current study where, during the chronic HS, calves fed the low forage ratio (4:6).

## 4. Materials and Methods

### 4.1. Animals and Climatic Chamber Details

Serum samples for metabolomics used in this analysis were obtained as described in a previously published study by Kim et al. [3]. Briefly, sixteen Hanwoo bull calves (169.6 ± 4.6 days old, BW of 136.9 ± 6.2 kg) were used. The animals were randomly distributed into four homogenized groups (four calves each), namely thermoneutral (CON, temperature–humidity index: THI 68 to 70), mild (T1, THI = 74 to 76), moderate (T2, THI = 81 to 83), and severe (T3, THI = 88 to 90) stress groups placed into climate-controlled chambers. Two animals were housed in each chamber (2.5 × 2.5 × 3 m^3^, length, width, and height, respectively) at a time. A total of four chambers were operated (two chambers per treatment).

**Figure 4:**
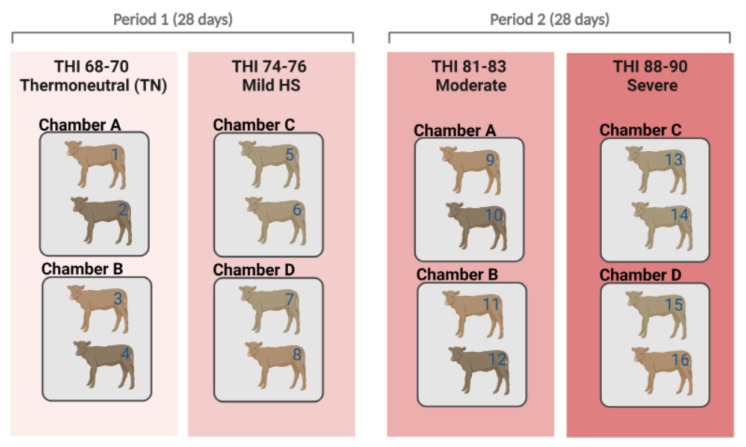
Schematic representation of climate chamber experiments according to heat stress (HS) treatments (thermoneutral, mild, moderate, and severe). Sixteen Hanwoo calves were randomly divided into four homogenized groups. The animals were subjected to ambient temperatures (22 °C) for 7 days in the adaption period one week before the experiment began. Following the adaptation periods, animals were kept in the treatment chambers for 21 days. After the completion of two treatments (thermoneutral and mild HS), we conducted the other two treatments (moderate and severe HS), using the same approach with different calves. THI = temperature–humidity index.

After completing two treatments, the other two treatments were carried out using different calves. Hence, four calves were first subjected to the threshold treatment, and four other calves were subjected to the mild HS treatment. After finalizing these first two treatments, we conducted the other two treatments (namely, moderate and severe HS treatments) using the same approach with different calves [19] (Figure 4).

### 4.2. Management Conditions and Treatment

The ambient temperature (AT) and relative humidity (RH) in a chamber were set to maintain the THI treatments from 9:00 a.m. to 7:00 p.m. daily. During the night-time (from 7:00 p.m. to 9:00 a.m. daily), the THI was maintained under 68, which did not influence the physiological parameters of the animals [48]. The calves were subjected to AT (22 °C) for 7 days in the adaptation period one week before the experiment began. Following the adaptation periods, animals were kept in the treatment chambers for 21 days. The four THI treatments were defined as follows: (1) thermoneutral (22 to 24 °C, RH: 60%, THI = 68 to 70), (2) mild (26 to 28 °C, RH: 60%, THI = 74 to 76), (3) moderate (29 to 31 °C, RH: 80%, THI = 81 to 83), and (4) severe (32 to 34 °C, RH: 80%, THI = 88 to 90). The chamber AT and RH were recorded at intervals of 1 s using two sensors SHT7x (Sensirion AG, Staefa ZH, Switzerland). The THI was calculated based on the dry bulb temperature (T_db_, °C) and RH using the following formula: THI = (1.8 × T_db_ + 32) − [(0.55 − 0.0055 × RH) × (1.8 × T_db_ − 26.8)] adopted from the previous study [49]. The diets used in this study were composed of 40% roughage (*Phleum pratense* L.) and 60% concentrate. The feed was weighed and offered twice daily at 9:00 a.m. and 5:00 p.m. The chemical composition of the feed was shown in the previous study [50].

### 4.3. Blood Sample Preparation

Blood samples were collected into clot activator tubes (BD Vacutainer, Belliver Industrial Estate, Plymouth, UK) on days 7, 10, 13, 16, 19, 22, 25, and 28 (2:00 p.m.) relative to the trial initiation. The serum samples were obtained from the blood after centrifugation at 2700× *g* for 15 min at 4 °C. The serum was then transferred to 1.5 mL tube (Eppendorf AG, Hamburg, Germany) and kept at −80 °C for further analysis.

Extraction of the pooled serum of each calf was conducted by adding 450 µL of cold methanol to 150 µL of serum in an optimal ratio of 1:3. Each serum sample was homogenized (with a frequency of 30) using a mixer mill (Retsch GmbH & Co, Haan, Germany) for 10 min and stored at −20 °C for 1 h. After that, centrifugation of the sample was performed at 13,000 rpm for 10 min at 4 °C. The supernatants were then passed through an additional 0.2 µm PTFE filter and transferred to 1.5 mL tubes. The supernatant was dried with a speed vacuum machine. The dried serum sample was dissolved by methanol and syringe filtration (0.2 µm) prior to gas chromatography time-of-flight mass (GC-TOF-MS) analysis. For the GC-TOF-MS analysis, a two-step chemical induction was performed for each sample. An oxidation process was carried out with 50 µL of methoxamine hydrochloride (20 mg/mL in pyridine, 90 min, 30 °C), followed by silylation using 50 µL of N-Methyl-N-(trimethylsilyl) trifluoroacetamide (MSTFA) (30 min, 37 °C), using gas chromatography–mass spectrometry (GC-MS).

### 4.4. GC-TOF-MS Analysis

The prepared serum samples were analyzed by an Agilent 7890B GC system (Agilent Technologies, Santa Clara, CA, USA) and a Leco TOF Pegasus BT mass spectrometry (LECO, St. Joseph, MI, USA). A DB-5MS capillary column (30 m length, 0.25 mm i.d, 0.25 µm film thickness; J &W Scientific, Folsom, CA, USA) was used for a helium gas flow of 1.5 mL/min. Then, 1 µL of the sample was injected into the split mode (1:10) for analysis using the GC-MS analysis protocol. After 2 min of operation in an oven set to 75 °C, the temperature was increased to 300 °C, at a rate of 15 °C/min, and maintained for 3 min. In order to collect the electron ionization (EI) mode, mass data were collected using an EI method with an ionization energy of 70 eV and a mass scan range of 50–600 m/z at an acquisition rate of 20 spectra/s. The ion source temperature and injector were set at 230 °C and 250 °C, respectively.

### 4.5. Data Preprocessing and Statistical Analysis

GC-TOF-MS data were preprocessed using LECO Chroma TOF™ software (version 4.44; LECO Corp.); then, the data were converted into the NetCDF format (*.cdf) using LECO Chroma TOF™ software. After conversion, peak detection, retention time correction, and alignment were then processed with an online METALIGN software package (http://www.metalign.nl, accessed on 11 August 2021) [51]. The resulting data were exported to an Excel file. Multivariate statistical analysis was performed using SIMCA-P^+^ (version 12.0, Umetrics, Umea, Sweden). The data sets were auto-scaled (unit variance scaling) and mean-centered in a column-wise fashion. Unsupervised PCA was conducted to investigate the general aggregate state and to show the trends in different groups among all samples. PLS-DA was performed to compare each data set. The variables were selected based on the VIP value. Finally, the statistical difference between groups was assessed using analysis of variance (ANOVA) and Tukey’s honest significant difference (HSD) test in SAS 9.4 (SAS Institute Inc., Cary, NC, USA). Differences observed between means were considered significant at *p* < 0.05. We conducted an additional post hoc power analysis using G*Power (version 3.1.9.7, University of Düsseldorf, Düsseldorf, Germany) to verify the analysis of the difference between groups in this study. The post hoc power analysis was applied with α = 0.05, sample size = 16, and effect size = 0.56. The power of analysis (1 − β) for the difference among the groups was 0.82.

## 5. Conclusions

GC-TOF-MS technology enables a comprehensive analysis of metabolic profiles and can be used to identify potential biomarkers of metabolic disorders related to HS in calves. Twelve metabolites were identified as key potential biomarkers for recognizing HS status in Hanwoo calves. Moreover, the results presented in this study provide new insights into the metabolic pathways altered by HS. These metabolite analysis data were in close agreement with the results of the major stress parameters associated with metabolic changes in beef calves under HS conditions. This approach helps to identify the effective biomarkers and pathways involving in HS and to understand the basic mechanisms associated with HS in beef calves. However, further analysis of large-scale samples is warranted to validate the actual use of potential biomarkers and to explain the physiological mechanisms involved in the migration of HS-related metabolic pathways.

## Figures and Tables

**Table 1:** The candidate metabolites altered by heat stress as identified by gas chromatography–time-of-flight mass spectrometry (GC-TOF-MS) analysis.

Tentative Metabolite ^a^	RT ^b^	VIP	MS	Mass Fragment Pattern ^c^	TMS ^d^	ID ^e^
***Carbohydrates***						
Glycerol	7.25	0.72	205	45, 73, 103, 147, 205, 218	3	STD/MS
Ribitol	9.37	0.80	217	73, 103, 147, 217, 319	5	MS
Arabinose	10.54	0.94	307	73, 103, 147, 217, 307	4	MS
Ribose	10.75	1.91	103	73, 103, 147, 189, 217, 307	4	STD/MS
Arabitol	11.07	1.86	217	73, 103, 147, 205, 217, 307	5	MS
Sorbose	12.18	1.05	307	73, 103, 147, 217, 307	5	MS
Glucose	12.53	1.85	160	73, 103, 147, 205, 319	5	STD/MS
Myo-Inositol	13.62	1.65	305	73, 147, 191, 217, 305, 367	6	STD/MS
Galactose	13.94	1.52	319	73, 103, 147, 205, 319	5	STD/MS
Lactose	16.97	1.58	204	73, 103, 147, 204, 217, 361	8	STD/MS
***Amino acid***						
Alanine	5.52	0.78	116	73, 116, 147, 190, 218	2	STD/MS
Valine	6.69	1.46	218	73, 100, 144, 218, 246	2	STD/MS
Leucine	7.23	0.92	158	73, 147, 158, 218	2	STD/MS
Isoleucine	7.44	0.80	158	45, 73, 100, 158, 218, 260	2	STD/MS
Proline	7.49	1.08	142	73, 142, 216	2	STD/MS
Glycine	7.57	1.37	174	73, 86, 100, 147, 174, 248	3	STD/MS
Serine	8.06	1.40	218	73, 100, 147, 204, 218, 278	3	STD/MS
Asparatic acid	9.44	1.19	232	73, 100, 147, 218, 232	3	STD/MS
Methionine	9.45	1.55	176	61, 73, 128, 147, 176, 293	2	STD/MS
5-Oxoproline	9.49	0.84	156	73, 147, 156, 230, 258	2	STD/MS
Phenylalanine	10.33	1.19	218	73, 100, 147, 192, 218, 266	2	STD/MS
Asparagine	10.65	1.95	116	73, 116, 132, 231	3	STD/MS
Ornithine	11.72	0.71	174	73, 142, 174, 200, 420	4	MS
Lysine	12.45	2.26	317	73, 92, 128, 174, 230, 317	4	STD/MS
Tyrosine	12.58	1.34	218	73, 100, 147, 179, 218, 280	3	STD/MS
Tryptophan	14.35	0.93	73	45, 73, 202, 291	3	STD/MS
***Fatty acids***						
Oleic acid	14.18	1.64	339	73, 117, 129, 145, 339	1	STD/MS
Oleamide	15.32	0.87	75	75, 116, 128, 131, 144, 338	1	STD/MS
***Organic compounds***						
Acetic acid	5.24	1.37	147	45, 66, 73, 147, 177, 205	2	MS
Urea	6.77	1.66	147	45, 73, 147, 171, 189, 204	2	MS
Butenedioic acid	7.87	1.84	245	45, 73, 147, 245	2	MS
Threonic acid	9.81	0.83	292	73, 117, 147, 205, 220, 292	4	STD/MS
Glyoxylic acid	14.90	1.01	203	75, 113, 147, 203	2	MS
***Nucleotides***						
Pyrimidine	7.86	1.93	241	45, 73, 99, 147, 241, 256	2	MS
***etc.***						
Pentanedioic acid	9.89	1.52	198	73, 147, 198, 304	2	MS
Propanedioic acid	11.12	2.25	305	73, 147, 221, 305	3	MS
Phosphoric acid	11.35	0.79	299	73, 103, 147, 357, 399, 445	4	MS
9, 12-Octadecadienoic acid	14.16	1.32	338	75, 81, 95, 117, 129, 337	1	MS

^a^ Identified metabolites based on variable importance projection (VIP) analysis with cutoff value at 0.7; ^b^ Retention time; ^c^ Mass fragmentation is the fragmentation of the tentative compound; ^d^ Trimethylsilyl; ^e^ Identification: MS, mass spectrum was consistent with those of NIST and in-house libraries; STD, mass spectrum was consistent with that of standard compound.

**Table 2:** List of potential metabolic pathways that change in serum according to heat stress.

Metabolite Pathway	*p*-Value	Impact	Metabolites
Phenylalanine, tyrosine, and tryptophan biosynthesis	0.042	0.5	Lysine
Aminoacyl-tRNA biosynthesis	0.001	0.17	Asparagine, Lysine
Galactose metabolism	0.002	0.16	Lactose, Galactose, Myo-Inositiol
Glyoxylate and dicarboxylate metabolism	0.043	0.04	Acetic acid
Glycolysis/Gluconeogenesis	0.029	0.03	Acetic acid
Inositol phosphate metabolism	0.317	0.13	Myo-Inositol
Pyruvate metabolism	0.210	0.06	Acetic acid
Phosphatidylinositiol signaling system	0.260	0.04	Myo-Inositol
Cystein and methionine metabolism	0.299	0.02	Asparagine, Lysine
Glycine, serine and threonine metabolism	0.306	0.21	Serine, Lysine
Tyrosine metabolism	0.364	0.14	Tyrosine

## Data Availability

Not applicable.
